# Communal sheep farmer’s knowledge and attitudes on the incidence of gastrointestinal parasites in the Eastern Cape, South Africa

**DOI:** 10.5455/javar.2022.i602

**Published:** 2022-09-29

**Authors:** Mlungisi Selby Jansen, Nkululeko Nyangiwe, Yanga Simamkele Diniso, Mandla Yawa, Thando Conference Mpendulo, Mzwethu Dastile, Ishmael Festus Jaja

**Affiliations:** 1 Dohne Agricultural Development Institute, Stutterheim, South Africa; 2 Department of Livestock and Pasture Science, University of Fort Hare, Alice, South Africa; 3 Department of Rural Development and Agrarian Reform, Cradock, South Africa; 4 Department of Agriculture and Animal Health, University of South Africa, Florida, South Africa

**Keywords:** Agro-ecological zones, farmer’s perception, gastrointestinal parasites, sheep

## Abstract

**Objective:**

Gastrointestinal parasites (GIPs) negatively impact small ruminant production and productivity nationwide, particularly in tropical and sub-tropic regions. Amongst other nematodes, *Haemonchus contortus*, *Trichostrongylus colubriformis*, and *Teladorsagia circumcincta* are the most common species in small ruminants animals. Thus, this study aimed to investigate communal sheep farmer’s knowledge and attitudes toward GIPs in the Eastern Cape Province, South Africa.

**Materials and Methods:**

A cross-sectional survey was conducted between September and November 2018 from three agro-ecological zones, namely, arid region, semi-humid, and humid. All data from this study were analysed with the Statistical Analysis System.

**Results:**

Of the total of 107 farmers who participated in the study, 69% were males, and 38% were females. Most livestock owners (85%) were aged >46 years old across all the study areas. The majority of farmers (83%) perceived that their animals are susceptible to wireworm (*H. contortus*) during the hot-wet season, followed by the hot-dry season (14%), with relatively low during the cold season (2%). Most farmers (85%) interviewed revealed that lambs are more exposed to parasitic infection, than mature sheep (15%) across all agro-eco­logical zones. An insignificant number of farmers (8%) with knowledge about GIPs life cycle and its biology (92%) across all agro-ecological zones. This study reveals a significant increase in the occurrence of GIPs over the past few years across all agro-ecological, with largely (67%) attributed to the resistance of the strain to deworming remedies and changes in climatic weather patterns (33%). The farming experience was strongly (*p* < 0.05) associated with the farmer’s gender and age. Helminths were reported significantly higher (*p* < 0.05) in humid zones than in other agro-ecological zones.

**Conclusions:**

This study concludes that most farmers perceived lambs as more susceptible to GIPs than old sheep. Therefore, farmers should be enlightened about the infection and transmission dynamics of the GIPs to develop appropriate control measures against worm infection to boost sheep production in the study area. It was also suggested that farmers should adhere to remedial instruction and adopt rotational deworming programs to avoid anthelmintic resistance.

## Introduction

Knowledge transfer in the livestock industry remains an important constraint to efficient production. Hence, stakeholders in the livestock sector need to find innovative ways to communicate modern scientific farming methods that are socially acceptable and ensure a market for animal-source food continues to thrive [1]. In many African countries, agriculture plays an essential economic role in improving many livelihoods [2]. Understanding specific limiting factors for emerging farmers is vital in preparing effective policies and development programs, strategies, and models that will enhance and support the transition of small-scale farmers into commercial farming [3]. Communal farmers are crucial stakeholders in rural development; however, their movement to urban areas negatively affects the strategy of rural revitalization implementation [4]. Moreover, it has been noticed that the farming experience and opinions of those who have long been involved in livestock farming have often been ignored. Many perceive their information as not valuable because it is not scientifically proven [5].

Livestock farming plays a crucial role in socio-cultural and economic well-being for many households, including agricultural diversification, income, food security, saving and employment, transport, soil fertility, traction, and sustainable agricultural production [6]. Infection by Gastrointestinal parasites (GIPs) is described as a major setback by many small ruminant farmers worldwide as it hinders production [7]. In South Africa (SA), infection by nematode parasites has been a largely notifiable challenge to many small ruminant farmers, attributed to their resistance to most commercial anthelmintics [8,9]. Many small ruminant farmers in SA from both resource-limited farming systems and commercial farming systems and in SA in the past have described *Haemonchus contortus* as the major challenge in their livestock production [10]; however, the winter season has been described with less infection rate [11]. Infections with GIPs compromise the animal’s health, resulting in clinical and sub-clinical diseases leading to financial loss, decreased production, and animal death [12]. Indiscriminate use of anthelmintic drugs to control GIPs results in anthelmintic resistance, a global challenge in sheep-producing countries, including SA [10].

Several studies address the challenges of small-scale farmers in Africa and maintain that nematodes and coccidia are highly affecting small stock production [9,13]. Therefore, the paper provides incidence, knowledge, and reports on the attitudes of communal sheep farmers on GIPs in the Eastern Cape Province (ECP), SA.

## Materials and Methods

### Ethical clearance

Permission to conduct this study was granted by the Animal Ethics Sub-committee (Ethical clearance certificate number 1/2012) of the Department of Rural Development and Agrarian Reform in the ECP, SA. All the participants were briefed about the aim of the study, and voluntary participation was encouraged.

### Study areas

The study was conducted between September and November 2018 in three agro-ecological zones in EC, SA.The study was conducted at Wartburg community (27° 25’56” E, 32°, 26’18” S; alt. 899 m), situated in Stutterheim on the sour veld (humid), and it is 899 m above sea level. It falls under Amahlathi local municipality in the Amathole District Municipality. The frost incidence is variable (7–65 days) but is higher in the northwest and under Tsomo Grassland [14]. Enoch Mgijima municipality (26° 47’19” E, 32°, 15’58” S; alt. 1,495 m) falls under Chris Hani District Municipality. Rainfall peaks later during the summer season. The mean annual precipitation is 380 mm west, increasing to 640 mm east. The incidence of frost of 22–58 days is higher in the northwest than in the southeast and under Queenstown thornveld [14]. Inxuba Yethemba is situated at 25° 41’28” E, 32°, 08’58” S; alt. 1,385 m in Chris Hani District Municipality. The rainfall is common in the autumn and summer and peaks in March. The average annual precipitation varies from 180 mm west to 430 mm east. The area is characterized by high frost incidence ranging from <30 days to 80> days per year [14].

### Data collection

30% of 107 structured questionnaires were prepared and used before the commencement of the study for the pre-sampling phase to get farmers’ inputs to ensure no useful information is left behind. After the pre-sampling phase, the study was conducted using face-to-face interviews with willing farmers during the data collection period using the vernacular Xhosa language. 30% sampling method was used to achieve the study’s objective. Before beginning the data collection, all participants were informed about the study objectives and were assured that their participation was voluntary and their information would be kept confidential.

A structured questionnaire consisting of closed, semi-closed, and open questions was used to collect information from the farmers. The questions were organized in different sections to collect socio-economic data and information regarding animal husbandry practices. The first section covered socio-demography such as farmer’s name, gender, age, level of education, and estimated monthly income. This was followed by livestock production characteristics such as the number of livestock species kept, the effect of internal, and management of grazing areas. In addition, questions were asked regarding helminth control methods, frequency of deworming in summer and winter, and whether or not the existing deworming compound is efficient. Farmers were also asked to indicate reasons for observing livestock internal parasite problems and rank them according to the severity from 1 (less severe) to 5 (very severe). The second part of the questionnaire referred to alternative measures for internal challenges and knowledge of internal parasites and parasites species that are commonly observed. The last section of the questionnaire focused on climate change’s impact on livestock production. At all times, farmers had the opportunity to clarify questions and were allowed to include their personal information or comments.

### Data analysis

Statistical Analysis System version 9.1 (2003) was employed to analyze all data collected from the farmers. Frequencies and percentages was present the result. The associations between farmers’ demography and their attitudes on gastrointestinal disease parameters, internal parasite control methods, and knowledge of worm resistance were achieved using the Chi (*χ*^2^)–square test.

## Results

### Smallholder farmers’ demography and socio-economic characterization

Smallholder farmers’ demography and socio-economic characterization are presented in Table 1. Of the 107 farmers participating in the study, 68% and 32% were males and females, respectively. Most livestock owners (85%) were aged >55 years from the three agro-ecological zones. A significantly higher percentage (49.53%) of the farmers have no formal education (illiterate), followed by farmers who had primary education (34. 58%) and the lowest (0.93%) having obtained degree qualifications. Most farmers (85.05%) have received agricultural-related training, and very few (14.95%) did not receive any training. A significantly higher number of farmers (95.53%) interviewed in all three agro-ecological zones were involved in farming activities, and the lowest number (4.67%) were not farming at all. The highest (85.05%) of farmers earn an income of between R500.00 (33.46 US$) to R2,000.00 (133.84 US$) per month, and very few (1.87%) make more than R10,000.00 (669.19 US$) per month. Most farmers (98.13%) are members of different agricultural organizations. A small percentage (1.57%) are not affiliated with any agricultural organizations or structures.

**Table 1.** Demographic information of sheep farmers in Amahlathi, Enoch Mgijima, and Inxuba Yethemba local municipality in the ECP.

Demographic	Frequency (*n* = 107)	Percentage%	*χ* ^2^	*p*-Values
**Gender**				
Female	38	35.51	*	0.042
Male	69	64.49		
Total		100		
**Age**				
36–45	16	14.99	**	0.002
46–65	91	85.05		
Total		100		
**Level of education**				
No formal education	53	49.53	*	0.035
Primary	37	34.58		
Matric	11	10.28		
Diploma	5	4.67		
Degree	1	0.93		
Total		100		
**Training**				
Yes	91	85.05	**	0.002
No	16	14.95		
Total		100		
**Income**				
<500	6	36.1	**	0.003
500–2,000	91	85.05		
2,000–5,000	8	7.48		
5,000>	2	1.87		
Total		100		

*χ*^2^—Chi-square test, Significant at * *p* ≤ 0.05 and ** *p* ≤ 0.001.

### Farming livestock animals

**Figure 1.**
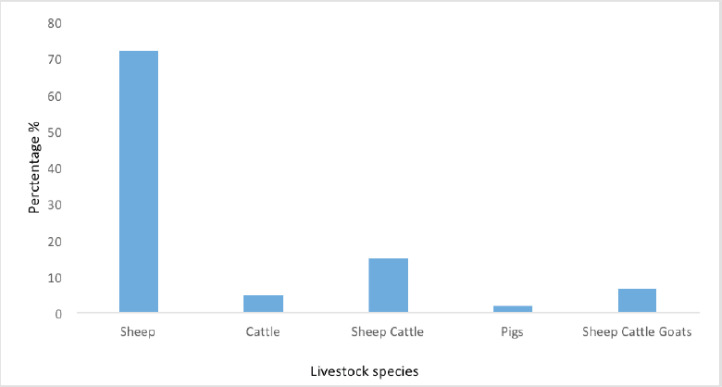
Farmer’s livestock species and numbers are kept by the communal farmers.

The highest number (70%) of respondents were sheep farmers, followed by sheep and cattle farmers (15%), mixed farming (8%) of sheep cattle and goats, others with cattle (5%) only, and the lowest number (2%) of farmers are involved in pig farming as shown in Figure 1.

### Seasonal occurrence of internal parasites

Table 2 shows farmers’ perception of the seasonal occurrence of internal parasites and animal age group infections. Roundworms (*H. contortus*) (97%) were the most affecting farmers, followed by tapeworm (2%), and coccidia had the least (1%) effect and significantly differed (*p* < 0.05). Most farmers (85%) noticed sick animals by bottle jaw signs. A significant difference (*p* < 0.05) was observed where about 85% of the farmers perceived that young sheep are the most affected group and least to 15% on mature sheep.

### Veld management

Table 3 shows farmer’s perception of grazing management practices and burning seasons. Most interviewed farmers (61%) in the study do not practice rotational grazing, and only (39%) of farmers practice rotational grazing. The highest of (64%) of farmers burn veld during the spring season and followed by (32%) of farmers who burn veld during late winter, and the lowest number (4%) burn during the autumn season. Very few farmers (5%) responded that they use their own bred breeding rams to face the challenge of wireworm resistance and the rest (95%) have no new alternatives to wireworm challenges. A significantly high number of farmers (92%) responded that they do not know about the life-cycle of internal parasites. Very few (8%) responded to understand the life-cycle of the internal parasites.

### Relationship between farmers’ demography and seasonal occurrence of parasites, life-cycle, and veld management

The association between demographic information and seasonal occurrence of parasites, parasite life-cycle, and veld management are shown in Table 4. The area significantly (*p* < 0.05) influences the animal species. The monthly occurrence of internal parasites was also significantly (*p* < 0.05) affected by farmers’ training. There was no significant difference (*p* < 0.05) between the parasite life cycle and demographic characteristics. The area significantly influenced rotational grazing and veld burning (*p* < 0.001). In addition, rotation grazing is influenced by the farmer’s level of education (*p* < 0.05). Climate change had no effect (*p* > 0.05) on the farmer’s education level and farmer’s training.

**Table 2.** Farmers perception on the seasonal occurrence of internal parasites and animal age group infections.

Demographic	Frequency (*n* = 107)	Percentage%	*χ* ^2^	*p*-values
**Parasites**				
Roundworms	104	97.20	**	0.001
Coccidia	1	0.93		
Tapeworm	2	1.87		
Total		100		
**Season**				
Winter	2	1.87	**	0.004
Spring	88	82.24		
Summer	16	14.95		
Autumn	1	0.93		
Total		100		
**Sheep age group**				
Young	91	85.05	**	0.002
Mature	16	14.95		
Total		100		

*χ*^2^*—*Chi-square test, Significant at * *p* ≤ 0.05 and ** *p* ≤ 0.001.

**Table 3.** Perception of farmers on grazing management practises and burning seasons.

Items	Frequency (*n* = 107)	Percentage (% )	*χ* ^2^	*p*-values
**Rotational grazing**				
Yes	42	39.25	NS	0.054
No	65	60.75		
Total		100		
**Veld burn season**				
Autumn	5	4.67	**	0.004
Winter	34	31.78		
Spring	68	63.55		
Total		100		
**Parasites challenge**				
Yes	5	5	**	0.001
No	102	95		
Total		100		
**Knowledge of internal parasites**				
Yes	98	91.59	**	0.001
No	9	8.41		
Total		100		

*χ*^2^*—Chi-square* test, Significant at ** *p* ≤ 0.001 and NS - not significant at *p* ≥ 0.05.

## Discussion

The current study reveals that livestock farming is the primary source of income for most rural household communities. The results align with Mmbengwa et al. [15], who reported that communal livestock farming is mainly practiced by rural households in low and middle-income countries, especially in Africa. Smallholder farmers’ demography and socio-economic characterization are crucial to understanding as indicators for sustainable agricultural [16]. The highest proportion (85%) of livestock was kept by farmers aged >55 years old and age <36 years old, respectively. The study suggests that the participation of youth in agricultural activities is less when compared to older people. A similar study found that youth would instead relocate to cities in search of job opportunities and earn better salaries, which makes them less involved in agriculture [17]. On the other hand, migration has also been associated with a survival strategy by the poor, especially in rural communities, due to fewer opportunities [18].

**Table 4.** Association between demographic information and educational level, farming training and seasonal occurrence of internal parasites.

Demographic	Animal species	Signs	Age of animal	Parasite name	Month of occurrence	Rotational grazing	Cause of parasites	Life-cycle	Veld burn	Climate change
Area	0.0123*	0.2765^NS^	0.4729^ NS^	0.1401^ NS^	0.3608^ NS^	0.0001**	0.0001**	0.1092^NS^	0.0001**	0.5852^NS^
Sex	0.469^NS^	0.0606^NS^	0.4554^ NS^	0.2325^ NS^	0.2344^ NS^	0.0002**	0.0009*	0.3839^NS^	0.0006**	0.2385^NS^
Age	0.5725^NS^	0.9727^NS^	0.0001**	0.7624^ NS^	0.2323^ NS^	0.3398^ NS^	0.0254*	0.1062^NS^	0.1837^ NS^	0.4513^NS^
Education	0.9072^NS^	0.7008^NS^	0.0982^ NS^	0.0071*	0.8397^ NS^	0.0325*	0.0163*	0.9058^NS^	0.0544^ NS^	0.9672^NS^
Training	0.2341^NS^	0.6804^NS^	0.2892^ NS^	0.7624^ NS^	0.0273*	0.4772^ NS^	0.8461^ NS^	0.7356^NS^	0.7977^ NS^	0.9578^NS^
Profession	0.9842^NS^	0.7071^NS^	0.5611^ NS^	0.0001**	0.7692^ NS^	0.3666^ NS^	0.7486^ NS^	0.3390^NS^	0.7056^ NS^	0.9578^NS^
Income	0.9840^NS^	0.6804^NS^	0.5611^ NS^	0.0082*	0.5458^ NS^	0.8227^ NS^	0.0212*	0.1527^NS^	0.8200^ NS^	0.0429*
Association	0.7215^NS^	0.9508^NS^	0.5494^ NS^	0.9710^ NS^	0.9319^ NS^	0.2511^ NS^	0.8192^ NS^	0.6653^NS^	0.5574^ NS^	0.9892^NS^

Significant at * *p* ≤ 0.05, ** *p* ≤ 0.001 and NS not significant at *p* ≥ 0.05.

The study shows suggest a higher proportion (50%) of the farmers did not receive a formal education, followed by farmers who had primary education (35%) and the lowest (1%) of having obtained degree qualifications. Most of the rural communities in the province’s rural areas have limited access to formal education, as attributed by Myeni et al. [16], where most livestock farmers had less knowledge about farming systems due to the lack of education. Rural communities have always remained far behind in education compared to counterparts in urban areas due to various factors such as connectivity and poor infrastructural conditions [19]. The relevance of education in agricultural expansion has been broadly affirmed, and education enhances the farmers’ farming skills and productive capabilities [20]. At the same time, skills development through education and training has always been a powerful lever for improvement [21]. The study results show that the highest number (85%) of farmers are earning an income of between R500.00 (33.46 US$) to R2,000.00 (133.84 US$) per month, mostly from old age government pension funds and social grants. The findings of a Namibian study support the results, which reveal that pensions in communal areas were the main source of revenue for the highest proportion of all households (33%), followed by wages (23%) and brewing (10%) [22]. One study reported that most of the income is generated by beef cattle, dairy cattle, goats, sheep, and chickens in Africa [23]. Thus, many households depend on social grants were the only and primary source of income for most rural households in Ngqushwa Municipality in the ECP [24].

The current research findings show that the highest number (70%) of farmers are farming with sheep in the ECP. The results are supported by the figures of Statistics of SA (2016), which says that the ECP was the leading province regarding sheep farming in agricultural households. It accounted for 66% of all farming households that farmed 11 to 100 sheep as well as 36% of those that farmed over 100 sheep [25]. The number of livestock in communal and small-scale settings is significant [26]. Still, these sectors need a rapid translation into commercialization to mitigate poverty and contribute to South African gross domestic product. The study suggests that most farmers (97%) use Anthelimntics to control GIPs infections in sheep, and very few farmers (3%) use medicinal plants. In one South African study, researchers reported the common use of commercial drugs to control parasites; however, most resource-poor farmers cannot afford them due to their expensiveness [27].

Moreover, it has been noticed that many livestock owners generally prefer to use medicinal plant knowledge to treat their animals rather than rely on traditional healers [28]. The study’s findings disagree with a study conducted elsewhere [29]. They reported that it had been estimated that close to 75% of poor farmers in the ECP administer traditional medicine to treat animal diseases.

Current research findings show thatmost farmers (85%) noticed sick animals by bottle jaw signs and if it warrants deworming. The study’s findings are inconsistent with those of [3], who reported that most households could not tell if the animal was sick or not and could not diagnose or apply first aid service before the condition became more severe. The study’s findings show that very few farmers (5%) responded that they use their own bred breeding rams to face the challenge of wireworm resistance. Also, communal farmers had inadequate livestock breeding knowledge [3].

Most of the interviewed farmers were uncertain if they knew much about the internal parasites and the diseases they cause to their livestock. A significantly high number of farmers (92%) responded that they do not know about the life-cycle of internal parasites. Very few (8%) responded to understand the life-cycle of the internal parasites. These findings are supported by studies conducted elsewhere, which reported that the local knowledge of diseases in communal areas is restricted [30]. A significant difference (*p* < 0.05) was observed where about 85% of the farmers perceived that young sheep are the most affected group and least to 15% on mature sheep. This agrees with the findings of one South African study, which reported that farmers perceived 89% of endo-parasites as the significant cause of death in young animals [31].

The study reveals that all farmers (100%) have reported that no new internal parasites have been seen over the past 20 years except those that currently affect their animals (Roundworms, tapeworm, coccidia, liver fluke, and nasal worm). Moreover, many developing countries within the southern hemisphere continue to experience a high incidence of global warming than in other countries of the continent [32]. The survey results show that an increased number of farmers (94%) were very concerned about the climate change effects on their livestock in all zones of the province, and most of the farmers (89%) agreed that climate change has a negative impact on their areas and the prevalence of the internal parasite. The effects of climate change on agricultural outputs and livestock are challenging to establish and distinguish from other changes in the human and natural environments [23].

## Conclusion

The study reveals that older people dominate the farming sector, and youth are less involved in agricultural activities. The current study results showed that most farmers are predominantly affected by GIPs, primary wireworms in SA. Very few farmers know the life cycle of the internal parasites. The highest prevalence of *H. contortus* may be due to its resistance to the available dewormers. Little is known about the breeding of wireworm resistance animals in many rural communities in the ECP. The changes in weather patterns are significantly associated with the increase in the number of GIPs.

## List of abbreviations

GIPs, Gastrointestinal parasites; ECP, Eastern Cape Province; SA, South Africa.
